# 

**Published:** 1871-02

**Authors:** 


### CONTENTS:

ORIGINAL COMMUNICATIONS:

ARTICLE I. Cases in Practice, with Remarks upon the Use of Veratrum Viride. By P. O. Reynolds, M.D.... 65

ART. II. Cases in Private Practice. By Jno. E. Owens, M.D.......... 75

ART. III. Obstinate Dyspepsia Cured by the use of Milk. By I. N. Danforth, M.D........................ 79

ART. IV. On a few Instruments for the Practical Treatment of Uterine Diseases. By Philip Adolphus, M.D................................ 81

ART. V. On some Points in the Operation for Hare-lip. Paper read before the Chicago Medical Society. By F. C. Hotz, M.D................ 88

ART. VI. Cases in Electro-Therapeutics. Reported by Wm. J. Maynard, M.D......................... 92

ART. VII. Cook County Hospital. Reported by Curtis T. Fenn, M.D.. 94

ART. VIII. Satan in Society. By a Physician. By VOLTA REEKE..... 108

SELECTION, AND COMMENTS........... 115

Opium in Metro-Peritonitis.

EDITORS' BOOK TABLE................. 120

Lectures upon Diseases of the Rectum.

BOOKS RECEIVED....................... 121

General Surgical Pathology and Therapeutics, in Fifty Lectures; On diseases of the Spine and Nerves; On the Wasting Diseases of Infants and Children; Body and Mind; Bartholow on Spermatorrhœa; Satan in Society; Circular No. 3—Approved Plans and Specifications for Post Hospitals; Circular No. 4—Report on Barracks and Hospitals, etc.

PAMPHLETS RECEIVED.................. 123

EDITORIAL............................ 124

